# Differences in exploration behaviour in common ravens and carrion crows during development and across social context

**DOI:** 10.1007/s00265-015-1935-8

**Published:** 2015-05-12

**Authors:** Rachael Miller, Thomas Bugnyar, Kerstin Pölzl, Christine Schwab

**Affiliations:** Department of Cognitive Biology, University of Vienna, Althanstrasse 14, 1091 Vienna, Austria; Haidlhof Research Station, University of Vienna and University of Veterinary Medicine, Bad Vöslau, Austria

**Keywords:** Corvid, Ontogeny, Neophobia, Exploration, Social context, Species differences

## Abstract

Exploration is particularly important for young animals, as it enables them to learn to exploit their surroundings. It is likely to be affected by species ecology and social context, though there are few comparative, longitudinal studies that control for effects of early experience. Here, we investigated group level exploration behaviour in two closely related and identically reared, generalist corvid species: common ravens (*Corvus corax*) and carrion crows (*C. corone*, *C. cornix*), during development and across social context. Subjects were repeatedly presented with a range of novel items, whilst alone and in a dyad/ subgroup, at the fledging (1–2 months old), juvenile (3–8 months old) and sub-adult (14–18 months old) stages. Whilst alone, they were also presented with a novel and familiar person, at the fledging and juvenile stages. We expected developmental differences and a facilitating influence of social context on exploration. Developmental differences were present, with both species interacting most frequently with novel items as juveniles, which may relate to major developmental steps, such as dispersal and a neophobia increase as sub-adults. When a conspecific(s) was present, subjects generally interacted more frequently, though took longer to interact, with novel items. Additionally, we found unexpected species differences, with the most striking difference being the crows’ significantly lower rate of interaction with the novel person, though not the familiar person; a species difference that was present from fledging. We discuss these findings by relating to potential differences in the two species ecology and behaviour, such as habitat use and caching proficiency.

Exploration through interaction with external stimuli, such as novel food and objects, provides individuals with opportunities to learn about and exploit their social and physical environments (Reader and Laland 2003). It is likely to be beneficial for individuals to be able to optimise on their exploration behaviour, for instance, in order to find sufficient food whilst avoiding predation risks. This exploration behaviour is particularly important to certain species, such as those that disperse, utilise different habitats or diets (Reader and Laland 2003). In a large comparative parrot study, for example, exploration (latency to touch a novel object) varied depending on the natural ecological conditions of each species (Mettke-Hofmann et al. 2002). Exploration may also be more important at specific life stages, such as in young animals. Age differences in exploration have been demonstrated in several species: in callitrichid monkeys, exploration is positively correlated with age (Kendal et al. 2005); in other species, it is the young individuals that are more investigative of novel items (Japanese macaque, *Macaca fuscata*: Menzel 1965; Australian magpies, *Cracticus tibicen*: Pellis 1981; vervet monkey, *Cercopithecus aethiops sabaeus*: McGuire et al. 1994), which may reflect greater playfulness (Greenberg and Mettke-Hofmann 2001).

Response to novel stimuli can produce not only curiosity, but also hesitance (‘neophobia’), which can be referred to as neophobic propensity. This co-existence of two behavioural responses, which has been reported in several species, is likely to enable animals to explore their environment and exploit new resources safely (Greenberg and Mettke-Hofmann 2001). The relationship between exploration and neophobia is influenced by the cost-benefits of the particular context. Species living with unpredictable dangers, such as high predation, may display lower exploration and higher neophobia. Higher exploration is likely to be beneficial when individuals need to gain new information about potential resources, such as in complex, variable habitats—like generalist species—or hidden food sources (Greenberg and Mettke-Hofmann 2001). In these cases, displaying a simultaneous high neophobic response—i.e. increased caution when exploring novelty—may serve to reduce the potential costs of high exploration (Greenberg and Mettke-Hofmann 2001).

Exploration, and other behavioural tendencies like neophobia, may also vary between individuals of the same species across differing environments (Reale et al. 2010). Trinidadian guppies (*Poecilia reticulata*) living in high predation pressure environments were found to be more ‘tenacious’ (ratio of feeding rates in presence and absence of a predator stimulus) than those living with lower predation risk (Fraser and Gilliam 1987). Common mynah (*Acridotheres tristis*) living in highly urbanized environments display lower neophobia compared with those in less urbanized settings (Sol et al. 2011). Exploration can therefore differ between species as well as within species, in response to age or environment.

Exploration of novel items is facilitated by the presence of conspecifics in several species. For instance, zebra finches (*Taeniopygia guttata*) will feed more quickly in a flock rather than alone (Coleman and Mellgren 1994). Capuchin monkeys (*Cebus apella*) will increase their acceptance of novel food after witnessing group members eating food (Visalberghi and Addessi 2001). A similar pattern has been found in young children, who will more readily eat novel food after observing peers eating that food (Birch 1980). In common ravens (*Corvus corax*), the presence of a sibling has been found to increase latency to approach novel objects, though it additionally increases the frequency of manipulation, at 3 and 6 months of age (Stowe et al. 2006). However, it remains unknown in this species when this pattern may first appear during ontogeny, whether it persists into later life, applies to other type of novel items like food or structures, and is apparent in other closely related species.

Here, we took an integrative approach and investigated the exploration behaviour of two closely related corvid species—common ravens and carrionx hooded crows—towards different types of item categories across ontogeny. By appearance, the crows were hybrids of the carrion crow (*Corvus corone*) and hooded crow (*C. cornix*), reflecting the hybridization belt in Europe. However, we will refer to the crows as ‘carrion’ hereafter, as carrion and hooded crows have highly similar life histories, and are usually considered to be subspecies (Glutz et al. 1993). The life histories of common ravens and carrion crows are highly likely to promote exploration behaviour, particularly during development. They are opportunistic generalists that utilise a range of habitats, though generally only the crows are found in highly populated urban environments (Bent 1946; Goodwin 1976; Boarman and Heinrich 1999). Both species have flexible social structures, based on fission-fusion dynamics with seasonal variation (Richner 1990; Braun et al. 2012). As juveniles, they appear to be relatively neophilic, whilst as adults, both species are highly neophobic (Kilham 1990; Heinrich 1995). In ravens, the increase in neophobia appears to take place within their first year as, in one study, juvenile ravens quickly contacted novel food and objects, though this reduced or stopped altogether by the time they were sub-adults (Heinrich 1995). However, the way in which this neophobia increase may affect exploration behaviour in ravens in relation to other stimuli, such as a familiar and novel person, and an assessment of whether these findings also apply to carrion crows, is still to be determined.

Our study aimed to address three main questions relating to group-level patterns of exploration behaviour in ravens and crows. First, we expected that behaviour towards novel food, (small and movable) objects and (large and non-movable) structures, as well as a novel and familiar person, would be influenced by life stage, which we classified as fledging (1–2 months old), juvenile (3–8 months old) and sub-adult (14–18 months old). These life stages were selected to adequately encompass several important steps that occur during development in these two species. At the fledging stage, the young birds are dependent on their parents and reside within family groups. As juveniles, the birds will become independent from their parents, join non-breeder flocks and disperse from their natal territories. As sub-adults, they are already members of a non-breeder flock with specific relations in which they engage, such as searching for a pair partner (Cramp and Perrins 1994; Ratcliff 1997).

We expected to see the clearest age differences within the time period when the birds are likely to become more neophobic, from the juvenile to sub-adult stage. In particular, we expected the highest rate of interaction with novel food to occur at the juvenile stage, when it is necessary for the birds to begin sourcing their own food, rather than relying on their parents. Similarly, we expected novel object interactions to increase at the juvenile stage, when the birds become more mobile, are exploring potential food sources, developing caching skills and potentially utilising objects in social interactions (Bugnyar et al. 2007a, b). We also included a novel structure condition, as, alongside exposure to novel food and objects, these birds are likely to encounter new structures in their environments that are often of anthropogenic origin. This is particularly true when they disperse during the juvenile stage, when we expected novel structure interactions to be highest. Furthermore, unlike the small transferrable objects, we expected strong neophobic responses towards these large, non-movable structures in older birds. Adult ravens and crows are renowned for their high degree of neophobia, which has been proposed to be linked with the feeding niche, as, unlike some other corvid species, they are scavenging specialists (Heinrich 1995; Chiarati et al. 2012). Ravens in particular will utilise the presence of predators to gain access into carcasses, and elevated caution is likely to be beneficial in these cases (Heinrich 1988, 2011). In response to the person, we expected both species to interact more frequently with the familiar than novel person, and, as with the other stimuli, a higher interaction rate at the juvenile stage when the birds are likely to be less neophobic.

We included a direct comparison between common ravens and carrion crows in order to assess whether our findings were likely to be specific to ravens or potentially reflect more corvid general patterns. As the two species have similar life histories, and in our lab were reared under the same conditions, we did not expect to find many species differences within life stages in their behavioural responses to the items. Finally, in order to investigate the influence of social context on behaviour, the novel food, object and structures were presented to the birds whilst alone as well as with one or two (primarily sibling) conspecifics. In line with a previous common raven study on novel object exploration (Stowe et al. 2006), we expected that social context would also facilitate raven exploration behaviour in other contexts—namely with novel food and structures. As ravens and carrion crows generally have comparable social structures, social context is likely to be equally influential in both species. We therefore expected to find a similar facilitating effect of social context on exploration behaviour in carrion crows.

## Methods

### Subjects

Subjects were nine captive common ravens, of which 3 were female, and ten captive carrion crows, of which 6 were female. The ravens were sourced at 25–38 days old (fledging ~45 days) from zoos in Austria, Germany and Sweden. Five ravens were first generation in captivity (i.e. their parents were wild-born), and four ravens were second generation in captivity (i.e. their grandparents were wild-born). The crows were collected at 10–14 days old (fledging ~30 days) from wild nests in the Donaupark, Vienna, Austria. All ravens and crows were hand-reared in 2012 under the same conditions—using the same rearers, diet, feed and training schedule, amount of human contact, social holding and type of enclosures. They were housed in species groups at the Haidlhof Research Station (University of Vienna and University of Veterinary Medicine) in large, outside aviaries (total size ~680 m^2^). Connected to the aviaries, and accessible by the birds through 2-m-high doors, were four large, indoor, separate test compartments (~20 m^2^ per compartment), which contained the same type of substrate and branching as the main aviaries. Immediately after fledging, the subjects were trained using positive reinforcement techniques to be able to be individually separated within the test compartments, and all test participation was voluntary. Subjects were individually identifiable via coloured leg rings and were well habituated to people, including the experimenter (RM). Subjects were satiated prior to testing to control for any differences in individual motivation driven by hunger, were never food-deprived in general and had constant access to water.

### Procedure

Subjects were presented with five types of stimuli during separate 10-min tests: novel food, novel (small and movable) objects, novel (large and non-movable) structures and both a familiar and novel person. For clarity, throughout this manuscript, we will refer to the novel food, objects and structure conditions separately from the familiar and novel person conditions. All tests were conducted within the test compartments, which were identical for both species. The novel food, objects and structures were presented whilst the subject was alone (temporarily separated from the group—hereafter ‘individual’ context) as well as with one or two conspecifics (hereafter ‘social’ context). The familiar and novel person conditions were presented in the individual context only.

In the individual context, the novel food, object and structure conditions were repeated ten times per individual, i.e. resulting in a total of 10 test rounds, from fledging (1 month old) until the sub-adult stage (approx. 1.5 years old; Table 1). Testing started as soon as the birds fledged (left the nest and were capable of short flights) in May 2012. The first and second test rounds were considered to take place within the ‘fledging’ stage (1–2 months old, May–June 2012), and the following 6 test rounds, repeated every 3 weeks, within the ‘juvenile’ stage (3–8 months old, July–November 2012). Following this, the last two test rounds were run in June and September 2013, which were considered to take place within the ‘sub-adult’ stage (14–18 months old). For the social context, the total of 19 subjects were assigned to 5 (two bird) dyads and 3 (three bird) subgroups, which were of primarily sibling composition (only two subjects did not have siblings). Here, the novel food, object and structure conditions were repeated five times from fledging (1 month old) until the sub-adult stage (1.5 years old; Table 1). We conducted one test round at the fledging stage (1 month old, May 2012), three rounds at the juvenile stage (3–8 months old, July, October and November 2012) and one round at the sub-adult stage (18 months old, September 2013).

**Table 1:** Outline of experimental design, including specific items utilised within each test.  = tested (individual context tested in all 10 test rounds and social context tested in 5 rounds)

Test round number	Life stage	Date	Individual context	Food	Object	Structure	Social context	Food	Object	Structure	Person (novel & familiar, individual context)
1	Fledging	May 2012	✓	Mealworms: whole, chilled	Plastic cup: green	Plastic bird bath: brown	✓	Crickets: whole, chilled	Plastic cup: red	Plastic bird bath: brown	✓ 2 females
2	Fledging	June 2012	✓	Strawberries: whole	Plastic straw: green	Concrete block: grey					
3	Juvenile	July 2012	✓	Cat biscuits: whole, dry	Cardboard juice carton: green	Perspex box: clear					
4	Juvenile	July 2012	✓	Kiwi: whole	Plastic Lego block: red	Wooden box: white	✓	Pineapple: slices	Plastic Lego block: blue	Wooden box: white	✓ 2 males
5	Juvenile	August 2012	✓	Peanuts: whole	Plastic box: green & purple, butterfly shape	Plastic chair: blue					
6	Juvenile	September 2012	✓	Corn-on-the-cob: half	Sponge ball: yellow	Plastic box: purple					
7	Juvenile	October 2012	✓	Chicks: whole, deceased	Plastic bowling pin: red	Wooden table: red	✓	Rats: whole, deceased	Plastic bowling pin: green	Wooden table: red	✓ 2 females
8	Juvenile	November 2012	✓	Oranges: whole	Plastic ball: pink	Plastic bin: blue	✓	Khakis: whole	Plastic ball: gold	Plastic bin: blue	
9	Sub-adult	June 2013	✓	Kohlrabi: whole	Plastic box: blue, rectangle, transparent	Plastic laundry basket: black					
10	Sub-adult	September 2013	✓	Cauliflower: half	Plastic box: green, square, opaque	Plastic shoe rack: white	✓	Peppers: whole	Plastic box: yellow, square, opaque	Plastic shoe rack: white	

The novel foods comprised several different categories: fruit, vegetables, meat and biscuits, which differed between test rounds, though were stable in food category within test rounds (Table 1). For example, in test round 1, two types of chilled insects were presented to the birds: mealworms (*Tenebrio molitor*) in the individual context and crickets (*Acheta domestica*) in the social context. The novel objects comprised similarly sized, small plastic or wooden items, such as cups, straws, blocks, balls or boxes, which also differed between test rounds, and were the same types though differed in colour within test rounds. For example, in test round 1, the same type of plastic cup was used: a green cup in the individual context and a red cup in the social context. For both the food and object tests, the same number of items as subjects present was used (i.e. two items for two birds present), with items being presented in the centre of a wooden board (board in place outside tests) on the ground.

In comparison to the food and objects, which were all transferrable items, the structures were much larger, non-movable items, consisting of plastic, wooden or concrete blocks, boxes, tables and chairs (Table 1). Within each round, the same structure was used for both the individual and social context, placed on the ground, though the position of the structure within the test compartment varied between contexts. For example, in test round 1, in the individual context, the structure (brown plastic bird bath) was placed on the right side of the compartment, whilst in the social context, it was placed on the left side, and vice versa in the following test round. Only one structure was present per test, regardless of the number of subjects present.

Additionally, subjects were presented with a novel and a familiar person, in the individual context only, three times from fledging until the juvenile stage—once at fledging (1 month old, May 2012) and twice at the juvenile stage (3–7 months old, July and October 2012). The person conditions were not tested at the sub-adult stage, as the birds were beginning to show aggression towards the test person at the final (juvenile stage) test and we did not want to encourage this behaviour. During the novel and familiar person test, the person was asked to sit still and remain as unresponsive as possible, in the same position on the ground. At each test round, the person’s identity was kept constant within species, and person’s sex was constant within all individuals (Table 1). For example, in test round 1, the same female person served as a familiar person for all birds, whilst a different, unknown (to the birds) female person served as the novel person. All subjects were well habituated to each familiar person, who they encountered during experiments, observations and/ or keeper duties on a regular basis (several times per week), and had never previously encountered the novel person.

One condition (food, object, structure, novel person, familiar person) was run per day, in the morning, with the order of both condition and context (individual and social) counterbalanced across test rounds. For example, in test round 1 (May 2012), the condition order was as follows: structure, food, novel person, familiar person and object on separate days in the individual context prior to the social context (i.e. the day before). Whereas in round 4 (July 2012), the condition order was as follows: object, familiar person, food, structure and novel person, with the social context tested first. Tests lasted 10 min each, starting when the item was placed on the ground, and were videotaped (Canon HD camera Legria HF510). The nine ravens were tested in all conditions and test rounds. Ten crows participated in all conditions and rounds, except the first and final two object, food and structure rounds (round 1 and 2, 9 and 10) when only 8 crows were available for testing, as 2 crows were obtained slightly later than the others, and 2 crows died (unrelated accident and *Clostridia* infection, June and July 2013).

### Data collection and analysis

For each subject within each test, we recorded latency and frequency of four distinctive behavioural parameters: being in proximity (movement around item within 0.5 m^2^), approach (directed movement towards item within 0.5 m^2^), touch (less than 3 s) and manipulation (more than 3 s) with the experiment item. Within the two latency and frequency variables, these four parameters (proximity, approach, touch, manipulation) were highly correlated, using the intra-class correlation coefficient (above 0.7, *p* < 0.05) to test for consistency between parameters. We therefore calculated a mean value for each variable—average of all four parameters for (1) latency and (2) frequency per subject within each test—which were then referred to as latency to interact and frequency of interactions.

The use of a mean measure, rather than a single measure (e.g. latency to proximity), allowed us to adequately convey the occurrence of all types of interaction with the items. We checked all results using two single measures—latency and frequency to proximity and manipulate—and found the results to be largely comparable. We selected the latency to interact measure in order to investigate how quickly the subjects were able to overcome neophobia—i.e. when they first approached and interacted with the items. The frequency of interaction measure was selected to see how much the subjects then interacted with the items. We consider both measures and their interplay, i.e. high latency/low frequency, short latency/high frequency or any other combination, crucial for characterising an individual’s exploration behaviour. Therefore, all frequency measures were collected throughout the duration of the 10-min tests independent at which point in time the subjects started interacting with the item. Additionally, we recorded the frequency of flying back and forth behaviour (repetitive movement patterns in short time, generally across the roof area), which is likely to reflect a fear response.

Experiment videos were coded by RM and KP using Solomon Coder (version 14.01.14). For inter-rater reliability testing, the two coders scored 12 % of videos, comprising a selection including all conditions and both contexts. As we scored durations and occasions in continuously recorded behaviour rather than discrete categories, we used Spearman’s rank correlations (Martin and Bateson 1993) and found very high inter-rater reliability (*ρ* > 0.991, *p* < 0.001).

Non-parametric tests (SPSS 19) were run, as most variables had a non-normal distribution. Figures were created using *SPSS* and *Adobe Illustrator*. Within species, to test for developmental differences and to compare the individual with the social context, we ran Friedman’s ANOVAs, which were followed by Wilcoxon signed ranks tests if significant. Between species, to test for species differences within life stages, Mann-Whitney *U* tests were used. In between species comparison cases where the shape of the distribution of the two samples was not similar, we used two sample Kolmogorov-Smirnov tests, which, unlike the Mann-Whitney *U* test, do not make any assumptions in term of distribution (Siegel and Castellan 1988). All tests were exact and two-tailed, and Bonferroni corrections were applied in cases with multiple comparisons (in this case, α was set to 0.05/3 = 0.017).

### Ethical note

Permission to remove crow nestlings from wild nests was approved by the Magistrate of Vienna for Environmental Protection, application number MA 22-355/2012/4. The licence number from the Ministry for Science and Research for permission to keep animals at Haidlhof Research Station is as follows: BMWFW-66.006/0011-WF/II3b/2014 from 22 May 2014. The birds were housed in accordance with Austrian Law and local government guidelines, and remained in captivity after the completion of this study for further behavioural research. The study was purely observational and entirely non-invasive, and therefore was not classified as an animal experiment according to Austrian Law (§2. Federal Law Gazette No.501/1989). However, the study was reviewed and approved by the Internal Animal Welfare Board at the Faculty of Life Sciences, University of Vienna (2014.012).

## Results

### Response to novel food, object and structures in the individual context: life stages and species differences

**Fig. 1:**
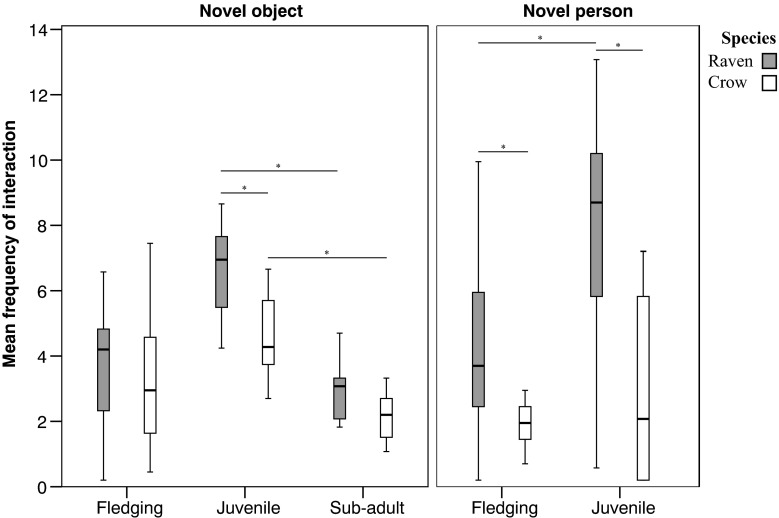
Frequency of interactions with *novel objects* and *novel person* in the *individual context* for both species. Significant differences between life stage and species are present. Plotted are median, quartiles and range. *Lines and asterisks* mark significant results of Wilcoxon signed rank tests and Mann-Whitney *U* tests with Bonferroni correction

*Within species*, there were significant differences in responses to novel food, objects and structures between the life stages. In the *object* condition, in comparing the fledging to juvenile stage, juvenile ravens showed a significantly shorter latency to interact with objects than when they were fledglings (Wilcoxon signed ranks test: *n* = 9, T^+^ = 45, *p* = 0.004). From the juvenile to sub-adult stage, both species interacted with objects significantly more frequently as juveniles than they did as sub-adults (Wilcoxon signed ranks test: *raven n* = 9, T^+^ = 45, *p* = 0.004; *crow n* = 8, T^+^ = 36, *p* = 0.008, Fig. 1). In the *food* condition, both species showed a significantly shorter latency to interact with food as juveniles than as fledglings (Wilcoxon signed ranks test: *raven n* = 9, T^+^ = 43, *p* = 0.012; *crow n* = 8, T^+^ = 36, *p* = 0.008). In the *structure* condition, as juveniles, both species showed a significantly shorter latency to interact with structures than when they were sub-adults (Wilcoxon signed rank test: *raven n* = 9, T^+^ = 45, *p* = 0.004; *crow n* = 8, T^+^ = 36, *p* = 0.008). Both species also interacted with structures significantly more frequently whilst they were juveniles than as sub-adults (Wilcoxon signed rank test: *raven n* = 9, T^+^ = 45, *p* = 0.004; *crow n* = 8, T^+^ = 36, *p* = 0.008).

*Between species*, there were significant species differences in responses to novel food, object and structures, primarily at the juvenile stage. In the *object*, *food and structure* conditions, juvenile ravens interacted with all items significantly more frequently than juvenile crows (*object*: Mann-Whitney *U* test: *U* = 16.5, *n*_1_ = 9, *n*_2_ = 10, *p* = 0.018, Fig. 1; *food*: Kolmogorov-Smirnov test: D = 0.6, *n*_1_ = 9, n_2_ = 10, *p* = 0.025; *structure*: Kolmogorov-Smirnov test: D = 0.7, *n*_1_ = 9, *n*_2_ = 10, *p* = 0.007; Table 2). In response to the *structure*, the crows showed a significantly higher frequency of flying back and forth behaviour than ravens as sub-adults (Kolmogorov-Smirnov test: D = 0.667, *n*_1_ = 9, *n*_2_ = 8, *p* = 0.011). Notably, the latter finding was the only significant species difference present at the sub-adult stage. Due to the presence of a contrasting sex ratio between species (raven: 6 males, 3 females; crow: 4 males, 6 females), which could confound the former results, we post hoc tested for sex effects and found no significant sex differences within any conditions.

**Table 2:** Response to novel food, objects, structures, person and a familiar person in the *individual context*. Significant differences present between life stages in response to food, objects, structures and the novel person for both species. Species differences in item exploration present, particularly at juvenile stage. Code: * = significant difference (*p* < 0.05, Bonferroni corrected), blank = no significant difference, ^ = higher values of measure

	Life stage	Measure	Object	Food	Structure	Novel person	Familiar person
Raven: Life stage differences	Fledging- Juvenile	Frequency				*Juvenile ^	
		Latency	*Fledging ^	*Fledging ^			
	Juvenile- Sub-adult	Frequency	*Juvenile ^		*Juvenile ^	N/A	N/A
		Latency			*Sub-adult ^	N/A	N/A
Crow: Life stage differences	Fledging- Juvenile	Latency		*Fledging ^		*Juvenile ^	
	Juvenile- Sub-adult	Frequency	*Juvenile ^		*Juvenile ^	N/A	N/A
		Latency			*Sub-adult ^	N/A	N/A
Species differences: Raven vs crow	Fledging	Frequency				*Raven ^	
		Latency					*Raven ^
	Juvenile	Frequency	*Raven ^	*Raven ^	*Raven ^	*Raven ^	
		Latency				*Crow ^	
		Flying back & forth				*Crow ^	
	Sub-adult	Flying back & forth			*Crow ^	N/A	N/A

### Response to a novel and familiar person in the individual context: life stages and species differences

There were significant differences in responses to the person within species across the life stages and between the novel and familiar conditions, as well as between species. *Within species*, the crows showed a significantly shorter latency to interact with the *novel person* whilst they were fledglings than as juveniles (Wilcoxon signed rank test: *n* = 10, T^+^ = 52, *p* = 0.01; Table 2). In comparing the *novel* and *familiar person* conditions, as juveniles, the crows showed a significantly shorter latency to interact with the familiar than the novel person (Wilcoxon signed rank test: *n* = 10, T^+^ = 54, *p* = 0.004). At both life stages, the crows also interacted significantly more frequently with the familiar person than the novel person (Wilcoxon signed rank test: *fledging n* = 10, T^+^ = 55, *p* = 0.002; *juvenile n* = 10, T^+^ = 52, *p* = 0.01). The only significant difference found for the ravens was with the *novel person*, where the ravens interacted significantly more frequently with the novel person as juveniles than as fledglings (Wilcoxon signed rank test: *n* = 9, T^+^ = 42.5, *p* = 0.019; Fig. 1).

*Between species*, for the *novel person* condition, the ravens interacted significantly more frequently with the person than the crows at both life stages (Kolmogorov-Smirnov test—*fledging*: D = 0.667, *n*_1_ = 9, *n*_2_ = 10, *p* = 0.014; *juvenile* D = 0.678, *n*_1_ = 9, n_2_ = 10, *p* = 0.016, Table 2, Fig. 1). In comparison with the juvenile crows, juvenile ravens also showed a significantly shorter latency to interact with the novel person (Kolmogorov-Smirnov test: D = 0.678, *n*_1_ = 9, *n*_2_ = 10, *p* = 0.018). As juveniles, the crows showed a significantly higher frequency of flying back and forth behaviour in the presence of the novel person than the ravens (Kolmogorov-Smirnov test: D = 0.6, *n*_1_ = 9, *n*_2_ = 10, *p* = 0.011). In the *familiar person* condition, there were no significant species differences in the frequency of interactions at either life stage (Mann-Whitney *U* test—*fledging*: *U* = 44, *n*_1_ = 9, *n*_2_ = 10, *p* = >0.05; *juvenile*: *U* = 34.5, *n*_1_ = 9, *n*_2_ = 10, *p* = >0.05). As fledglings, the crows did show a significantly shorter latency to interact with the familiar person than the ravens (Kolmogorov-Smirnov test: D = 0.689, *n*_1_ = 9, *n*_2_ = 10, *p* = 0.012). We post hoc tested for sex effects and found no significant sex differences within either condition.

### Response to novel food, object and structures: influence of social context

**Fig. 2:**
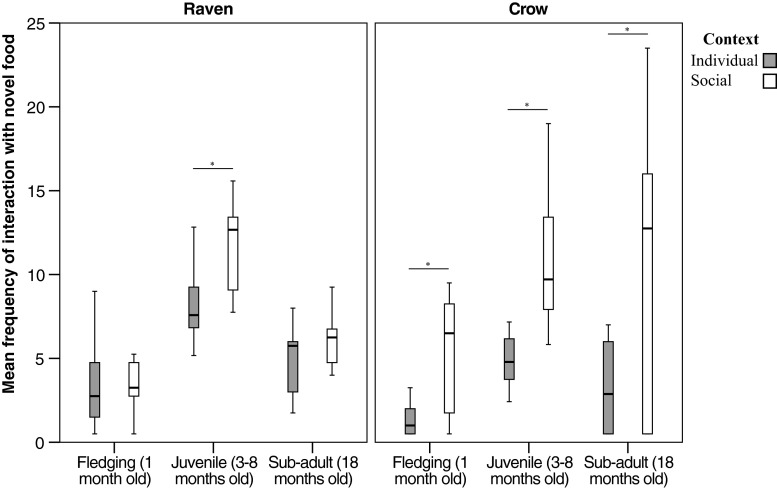
Frequency of interactions with *novel food* in the *individual* compared with *social context* for both species. Significant differences between individual and social context are present. Plotted are median, quartiles and range. *Lines and asterisks* mark significant results of Wilcoxon signed rank tests with Bonferroni correction

Both species showed significant differences in responses to novel food, object and structures in the social compared with individual context. In the *object* condition, sub-adult ravens and crows interacted with objects significantly more frequently when conspecifics were present than alone, with the crows also showing this pattern as juveniles (Wilcoxon signed ranks test—*raven*: *sub-adult*: *n* = 9, T^+^ = 45, *p* = 0.004; *crow*: *juvenile*: *n* = 10, T^+^ = 55, *p* = 0.002; *sub*-*adult*: *n* = 8, T^+^ = 35, *p* = 0.016; Table 3). In the *food* condition, as juveniles, subjects interacted with food significantly more frequently in the social compared with the individual context, with the crows showing this pattern as fledglings and sub-adults as well (Wilcoxon signed rank test—*raven*: *juvenile*: *n* = 9, T^+^ = 45, *p* = 0.004; *crow*: *fledging*: *n* = 8, T^+^ = 36, *p* = 0.008; *juvenile*: *n* = 10, T^+^ = 55, *p* = 0.002; *sub*-*adult*: *n* = 7, T^+^ = 28, *p* = 0.016; Fig. 2). In the food condition, sub-adult ravens and fledging crows showed a significantly shorter latency to interact with food whilst alone than when others were present (Wilcoxon signed rank test—*raven*: *n* = 9, T^+^ = 44, *p* = 0.008; *crow*: *n* = 8, T^+^ = 36, *p* = 0.008).

**Table 3:** Response to novel food, objects and structures in *individual* compared with *social context*. Significant differences in exploration present for both species whilst alone than with a conspecific(s). Code: * = significant difference (*p*<0.05, Bonferroni corrected), blank = no significant difference, ^ = higher values of measure

	Life stage	Measure	Object	Food	Structure
Raven: Social vs. individual context	Juvenile	Frequency		*Social ^	*Social ^
		Latency			*Individual ^
	Sub-adult	Frequency	*Social ^		
		Latency		*Social ^	
Crow: Social vs. individual context	Fledging	Frequency		*Social ^	
		Latency		*Social ^	
	Juvenile	Frequency	*Social ^	*Social ^	*Social ^
		Flying back & forth			*Individual ^
	Sub-adult	Frequency	*Social ^	*Social ^

In the *structure* condition, whilst they were juveniles, both species interacted with structures significantly more frequently when conspecifics were present than alone (Wilcoxon signed rank test—*raven*: *n* = 8, T^+^ = 35, *p* = 0.016; *crow*: *n* = 8, T^+^ = 35, *p* = 0.016). As juveniles, the ravens showed a shorter latency to interact with the structure when conspecifics were present than when alone (Wilcoxon signed rank test: *n* = 9, T^+^ = 44, *p* = 0.008). In the structure condition, as juveniles, the crows showed significantly more flying back and forth behaviour (fear response) in the presence of the structure, in the individual compared with social context (Wilcoxon signed rank test: *n* = 9, T^+^ = 45, *p* = 0.004).

## Discussion

In this study, we pursued an integrative approach to investigate group-level exploration behaviour, as determined by the latency and frequency of interaction with novel food, objects, structures and persons, in relation to life stage, social context and species, in common ravens and carrion crows. Within species, significant differences in exploration were present in responses to the novel food, objects and structures between the fledging, juvenile and sub-adult life stages though, as expected, they were primarily found at the juvenile stage. The decrease in exploration at the sub-adult stage is likely to reflect an increase in neophobia, which is supported by a previous study on food and object exploration in common ravens (Heinrich 1995). In addition to this previous study, we showed that this pattern is applicable to novel non-moveable structures as well, and holds in another corvid species, the carrion crow.

Exploring in a novel environment can be risky and can vary depending on the environment (Sol and Lefebvre 2000). Fast exploration can result in lower accuracy or higher risk behaviours, though can lead to short-term gains, such as access to food or mates (Sih and Giudice 2012). In some cases, however, high exploration and low neophobia can be advantageous, such as when most of the environment is unfamiliar or during establishment in a new environment. For example, invading house sparrows (*Passer domesticus*) are less neophobic towards novel foods than resident conspecifics (Martin and Fitzgerald 2005). Once established in a suitable new environment, displaying excess caution to novelty is likely to reduce risks, such as predation (Greenberg and Mettke-Hofmann 2001).

In ravens and crows, the juvenile stage encompasses the periods when the birds become independent and disperse from their parents’ territory (Cramp and Perrins 1994; Ratcliff 1997). During this stage, they will join a non-breeder flock and together start to explore new environments, which present a range of novel items, in order to locate suitable feeding and roosting areas (Marzluff and Heinrich 1991). As juveniles, it may therefore be beneficial to be quicker and more open to exploring new environments and food sources, in order to superficially sample the various potentially available options (Sih and Giudice 2012). By the sub-adult stage though, the ravens and crows are likely to be established within a non-breeder flock, which will generally feed and roost within familiar environments (Ratcliff 1997), and so, there may be less need for fast and frequent exploration (Sih and Giudice 2012). Our findings are therefore biologically meaningful, indicating that exploration and neophobia in these species are influenced by life history, which includes a change of physical and social environment due to dispersal and later territorial breeding.

Unexpectedly, significant species differences were present in responses to the novel objects and food, as juvenile ravens interacted more frequently with these items than juvenile crows. Both species are feeding generalists and should therefore be expected to be attracted to novel items in a similar way (Goodwin 1976; Boarman and Heinrich 1999). Object manipulation has previously been shown to play a role in the development of caching skills in juvenile ravens (Bugnyar et al. 2007a). Common ravens are proficient in their caching skills, for instance using visual barriers to avoid cache pilfering by conspecifics—a skill which first appears within the juvenile stage (6 months old; Bugnyar et al. 2007a). It may therefore be the case that caching, and as a consequence object interactions, may be of more importance to ravens than crows. The propensity and proficiency of caching in carrion crows in direct comparison with common ravens, however, are currently unknown.

Species differences were also present in relation to novel structures and the novel person, with the crows generally showing a higher fear response to these items. This finding speaks against the hypothesis that neophobia in corvids may be driven by the degree/amount of scavenging, as crows appear to be less reliant on carcasses than ravens (Bent 1946; Heinrich 1988). Compared with the ravens, the crows also took longer to interact and interacted less frequently with the novel, though not familiar, person. Previous studies show that wild American crows are able to differentiate between dangerous and neutral masked humans (Cornell et al. 2012). Additionally, adult carrion crows and common ravens have recently been shown to distinguish between human experimenters depending on their level of familiarity (Cibulski et al. 2013). Here, we show that, from the fledging age, carrion crows respond differently to novel and familiar humans, whilst the ravens respond in a similar manner to both person categories.

To our knowledge, this is the first study to demonstrate pronounced species differences between these two closely related species that are present at an early age (juvenile phase). These findings occurred despite the control factors, which include identical rearing from pre-fledging stage, holding and testing conditions. The ravens were sourced from captivity, whilst the crows were wild-born; however, this is unlikely to have driven these species differences, as the ravens were mainly first generation captive (i.e. their parents were wild-born). Furthermore, if species differences were driven by differences in source, we may expect to find more differences occurring from the beginning of the tests (i.e. from fledging), which we do not. Additionally, although the ravens reach sexual maturity later than the crows (ravens 3–4 years; crows 2–3 years old), their developmental steps, including independence and dispersal, occur at similar time points and within the same life stages (Cramp and Perrins 1994; Ratcliff 1997). We therefore feel that the life stages selected were comparable between species. Finally, although there were differences in sex composition of the two species, which could have potentially confounded the species differences, post hoc tests revealed no significant sex effects within any of the conditions.

Rather, these species differences relating to structures and novel persons may reflect propensities due to habitat use variation, as, unlike the ravens in general, crows regularly utilise urban habitats (Boarman and Heinrich 1999; Marzluff et al. 2001). Habitats contain different structures, with novel structures often being introduced by humans, and so, populated urban areas generally present a large range of novel structures. Crows may therefore be more likely to encounter novel structures during their early life, as they become independent and disperse, which may result in either a higher or lower neophobia—in this case, a higher neophobic response.

A strong negative response to new people may relate to hunting and persecution, which both species have experienced, both in some wild rural populations, as well as persecution particularly in the form of nest destruction in urban areas (Marzluff and Angell 2007; Amar et al. 2010). Corvids and other birds, such as European starlings (*Sturnus vulgaris*), will respond to differences in human behaviour towards them in urban areas (Clucas and Marzluff 2012). Indeed, Goodwin (1976) observed extreme corvid wariness towards changes in their environment in high persecution compared with low persecution areas. Urban living may lead to closer and more frequent interactions with humans, which are therefore potentially a threat; so, for urban-living crows in particular, such as those in the present sample, exhibiting high caution around unknown people may therefore be adaptive. On the other hand, urban-living species, due to increased exposure to humans, may also display decreased neophobia (e.g. common mynah; Sol et al. 2011), or increased aggression by territorial corvids in response to nest destruction by humans (Knight 1984). Our findings relating to exploration in these two corvid species, tested until sub-adulthood when they are not yet territorial, support the former rather than latter option.

Different types of items were used per condition for each test round, and test rounds were repeated a minimum of 3 weeks apart (in some conditions months apart), in order to reduce any possible habituation effect. If our results were related purely to a habituation effect, one would expect a decrease between the fledging to juvenile stage, or from the juvenile to sub-adult stage in all tests, which we did not find. Furthermore, the increased ‘fear response’ behaviour of the crows, though not the ravens, to the structure at the sub-adult stage, would suggest increased neophobia rather than simply decreased interest due to exposure.

It could also be suggested that our findings may be related to differences in the types of object, food and structures presented, such as a preference or aversion for a specific item, or to aspects of the person other than novelty, such as a positive association with the familiar person. Additionally, as crows are smaller than ravens, it could therefore be suggested that species differences, such as those relating to the structure and novel person, are related purely to this, or some other unknown difference in item perception. However, if this were the case, then one would expect to find significant differences both within and between species at all life stages, which was clearly not the case. On the contrary, we found that significant influences of life stage and species differences in exploration behaviour occurred primarily at the juvenile stage and differed depending on the type of stimuli presented, which we suggest may be related to important steps taking place within the natural life histories of these species at this time. Species differences, in these two closely related species with similar life histories, were unexpected, which highlights the benefits of this type of direct species comparison where early environment and rearing type have been controlled. It is not yet clear whether species differences may also occur later in life—follow-up studies may therefore aim to compare exploration behaviour in both species as adults.

Finally, we found that social context influenced both species in a similar manner. The presence of others delayed the time taken to interact with food in both species, which may indicate presence of ‘socially-induced neophobia’. Each individual may be waiting to allow the other(s) to take the risk of approaching first, or, if the other bird is higher in rank, they may have to wait for access (Marzluff and Heinrich 1991; Mainwaring et al. 2011). The presence of others also generally increased the frequency of interactions with novel food, objects and structures, and decreased the crows’ fear response behaviour to structures. It therefore appears that, once the birds have overcome this ‘socially-induced neophobia’, the presence of others results in an increased interaction and interest in the items, which may reflect social play behaviour (Diamond and Bond 2003). Indeed, object play may serve as a means of gaining information about conspecifics (Bugnyar et al. 2007b), or in forming and reinforcing social bonds (Bekoff 1984; von Bayern et al. 2007)—both of which would be important for these young birds living in social groups.

Previous studies have found that social context can either facilitate or inhibit exploration. Zebra finches have been found to feed more quickly in a flock than alone (Coleman and Mellgren 1994), though will also be less explorative of novel objects and environments while in a social context than alone (Mainwaring et al. 2011). It is likely that the items under exploration, e.g. food or objects, as well as the identity of conspecifics present, such as their rank, kin or affiliation relations to the focal, are important (Stowe et al. 2006). Here, we found presence of siblings to generally facilitate exploration of novel food, objects and structure during development.

Our social context-related findings are highly unlikely to reflect habituation across tests, as we counterbalanced the order of testing (individual vs social) and found not only an increase in frequency of interactions, but also an increase in latency to interact—a pattern not generally expected during habituation. The present study shows that these social context influences are also present in carrion crows, are applicable in both species to a range of novel items, specifically food, objects and non-movable structures, and are present from as early as fledging until at least 1.5 years old. It therefore appears that these findings may reflect a corvid-general pattern, rather than a raven-specific finding.
